# PET/MRI: a novel hybrid imaging technique. Major clinical indications and preliminary experience in Brazil

**DOI:** 10.1590/S1679-45082017MD3793

**Published:** 2017

**Authors:** Taise Vitor, Karine Minaif Martins, Tudor Mihai Ionescu, Marcelo Livorsi da Cunha, Ronaldo Hueb Baroni, Marcio Ricardo Taveira Garcia, Jairo Wagner, Guilherme de Carvalho Campos, Solange Amorim Nogueira, Elaine Gonçalves Guerra, Edson Amaro

**Affiliations:** 1 Hospital Israelita Albert Einstein, São Paulo, SP, Brazil.

**Keywords:** Positron-emission tomography/methods, Magnetic resonance imaging/methods, Tomography, x-ray computed/methods, Pelvic neoplasms, Prostatic neoplasms, Neuroimaging

## Abstract

In recent years, medical imaging with hybrid techniques has widely accepted and employed in clinical routine. PET/MRI offers significant advantages, including excellent contrast and resolution and reduced ionizing radiation, as compared to well-established PET/CT. Therefore, PET/MRI is a promising modality for oncologic imaging of some regions, such as brain, head and neck, liver and pelvis. This article set out to analyze clinical conditions that could benefit from PET/MRI imaging based on our caseload. The potential of PET/MRI to become the imaging modality of choice for assessment of neurologic and oncologic conditions associated with soft tissues is highlighted. Clinical aspects of PET/MRI and its application to clinical cases are illustrated with examples extracted from the authors’ preliminary experience.

## RESUMO

Nos últimos anos, imagens médicas com tecnologias híbridas tornaram-se amplamente aceitas e utilizadas na prática clínica. O PET/RM possui vantagens importantes, incluindo excelentes contrastes e resolução, e menor radiação ionizante, em comparação ao PET/TC. Por isto, é uma modalidade promissora para exames de imagem de pacientes oncológicos, para avaliar o cérebro, cabeça e pescoço, o fígado e a pelve. O objetivo deste artigo foi analisar as situações clínicas que se beneficiariam de exames de PET/RM a partir de uma casuística. Destacamos o potencial desta técnica se tornar o método de imagem de escolha para doenças neurológicas e oncológicas que envolvam partes moles. Os aspectos clínicos de PET/RM e sua aplicação aos casos clínicos são ilustrados com exemplos da experiência inicial dos autores.

## INTRODUCTION

Positron emission tomography (PET) was developed by Ter-Pogossian et al., in the 1970s, and implemented in clinical practice in the late 1980s and early 1990s.^1^

The first whole-body integrated PET/magnetic resonance imaging (PET/MRI) system has been recently introduced. The adoption of PET/MRI has been much slower compared to PET/computed tomography *(*PET/CT). Since its introduction in 2010, approximately 70 systems have been installed worldwide, mostly in universities.^2^ Equipment costs, operational costs and related logistics are thought to account for the slow adoption of the method. Furthermore, diagnostic benefits compared to well-established high performance modalities, such as PET/CT, are difficult to prove. Potential advantages of PET/MRI include high soft tissue contrast and functional MRI capability.^2^

This novel technology can provide simultaneous anatomic and molecular information and may come to achieve similar success to PET/CT, particularly in oncologic conditions, in which MRI is more indicated than CT due to improved soft tissue contrast. Magnetic resonance imaging is the current imaging modality of choice for oncological conditions associated with soft tissues (*e.g*., brain, head and neck, liver and pelvis).^3^

The aim of this paper is to describe and evaluate clinical aspects related to the use of PET/MRI in oncologic and neurologic diagnosis, and to illustrate the application of this imaging modality with examples extracted from the authors’ caseload and preliminary experience.

## METHODS

Five clinical cases involving patients referred to our institution for PET/CT are described in this article. Patients were submitted can PET/MRI immediately after PET/CT, without any further intravenous contrast agent injection for improved lesion characterization. Image acquisition was performed between 60 and 90 minutes, after intravenous injection of 148 to 333MBq of specific radiotracers (^18^F-FDG, Fluorodeoxyglucose or ^68^Ga-PSMA, prostate-specific membrane antigen labeled with ^68^Ga). Scanning time ranged from 30 to 90 minutes; images with high anatomical resolution and excellent soft tissue contrast were obtained.

### PET/MRI

PET/MRI with attenuation correction were acquired using dual echo VIBE Dixon or ultrashort echo time sequences for water and fat or bone separation. Anatomical MRI sequences were selected according to the type of exam (whole-body, head and neck, female and male pelvis or neurologic protocols). Simultaneous acquisition of PET images was performed using the following parameters: 500mm FOV, 400mm anterior-posterior FOV, 1.0 zoom, 3 interactions, 21 subsets, HD PET reconstruction method, and 2.0mm Gaussian filter (oncological assessment); or 300mm FOV, 300mm anterior-posterior FOV, 1.0 zoom, 8 interactions, 21 subsets, HD PET reconstruction and 2.0mm Gaussian filter (neurological assessment).

### Whole-body PET/MRI in oncology

**Figure 1:**
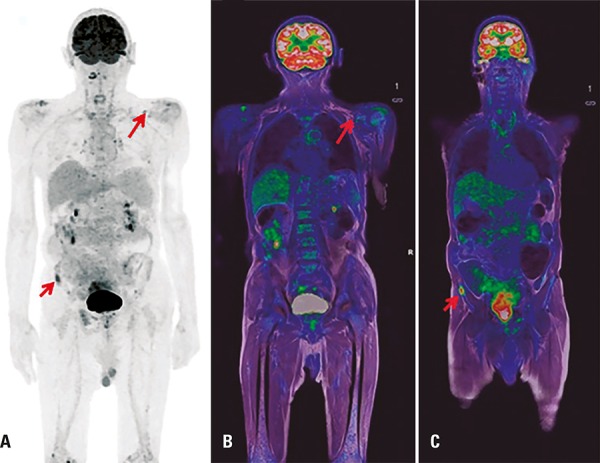
Post-chemotherapy whole-body 18F-FDG PET/MRI images of a male patient with multiple myeloma, showing residual metabolic activity in the right iliac crest and hypermetabolic left supraclavicular lymph node (arrows). (A) PET, maximum intensity projection image; (B and C) fused PET/MRI image, coronal T1

Whole-body PET/MRI is particularly indicated in cases requiring both the advantages of PET in detecting extramedullary disease, and the superiority of MRI in detecting spinal cord compression, extramedullary disease and active post-treatment residual sites. Therefore this novel technology is an attractive alternative for multiple myeloma and bone metastasis assessment^4^ (Figure 1).

### Head-and-neck PET/MRI

The role of PET/MRI in head and neck cancer diagnosis was first investigated by Boss et al.^5^ This imaging modality is attractive due to superior soft tissue contrast and the lower susceptibility of MRI to artifacts generated by dental metallic implants compared to CT.^6^

**Figure 2:**
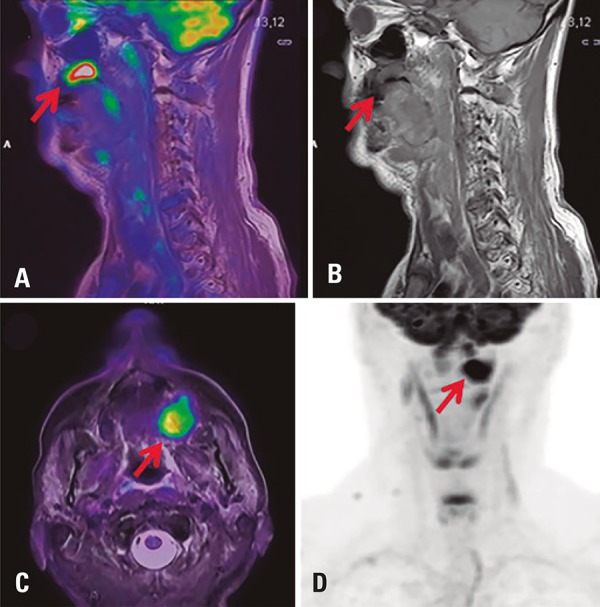
Head-and-neck 18F-FDG PET/MRI images showing hypermetabolic uptake in the left anterior portion of the gum line, extending to adjacent structures. (A) fused PET/MRI image, sagittal T1; (B) sagittal T1; (C) fused PET/MRI image, axial T2 fat saturated; (D) PET, maximum intensity projection image (arrows)

High spatial resolution and contrast of MRI are vital for tumor and regional lymph node staging in this complex anatomical region, given the ability of the method to delineate the actual tumor extent and distinguish lymph node involvement from surrounding normal tissue. Also, PET/MRI can be useful to detect distant metastatic spread, thereby contributing to radiation therapy and preoperative treatment planning (Figure 2).

### PET/MRI of the female pelvis

**Figure 3:**
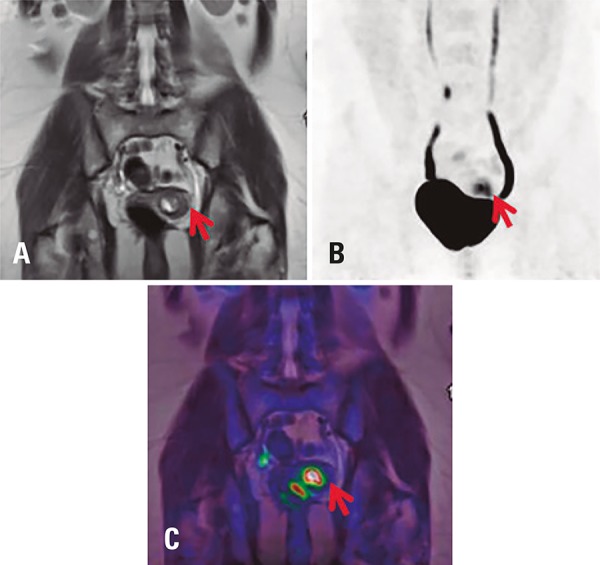
18F-FDG PET/MRI images of a female pelvis with invasive adenocarcinoma of the cervix showing metabolic activity in the left uterine wall, near the uterine cavity. (A) Dixon VIBE, coronal T2; (B) PET, maximum intensity projection image; (C) fused PET/MRI image, coronal T2

According to the National Comprehensive Cancer Network (NCCN) guidelines, PET/CT and PET/MRI are indicated as supplemental exams in cases suspected of gross cervical involvement or extrauterine disease.^7^ PET/MRI provides higher diagnostic confidence for discrimination between benign and malignant lesions compared to PET/CT (Figure 3).

### PET/MRI of the male pelvis

**Figure 4:**
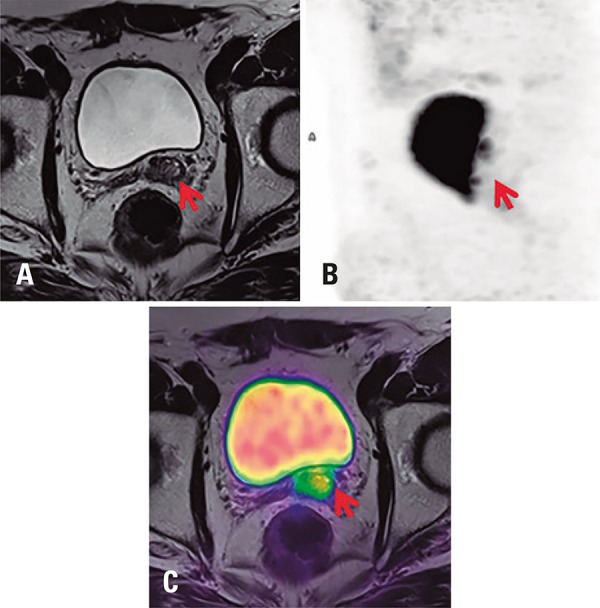
68Ga-PSMA PET/MRI image of a pelvis with recurrent prostate carcinoma in left seminal vesicle remnant, and marked PSMA expression in the lesion. (A) MRI image, axial T2; (B) PET, maximum intensity projection image; (C) fused PET/MRI image, axial T2

Multiparametric MRI (mpMRI) can be combined with ^68^Ga-PSMA PET for recurrent prostate cancer detection, staging and assessment. Superior results have been reported in the evaluation of the prostatic bed compared to PET/CT^8^ (Figure 4).

### PET/MRI in neurology

**Figure 5:**
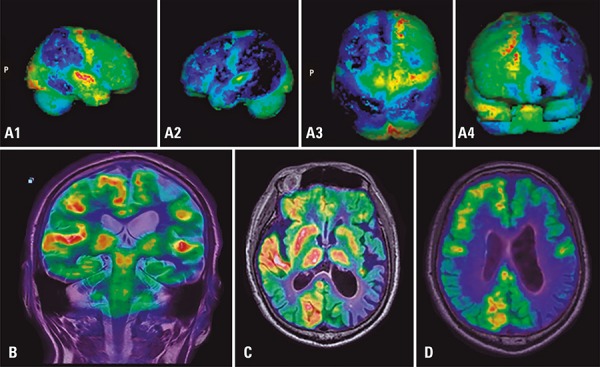
Brain 18F-FDG PET/MRI images of a male patient with Parkinson’s disease and cognitive impairment, showing glucose metabolism *deficit* and volumetric reduction in the left cerebral hemisphere. Similar findings can be seen in Lewy body dementia. (A1, A2, A3, A4) PET, volumetric reconstructions; (B) coronal T2; (C and D) fused PET/MRI images (FLAIR)

Magnetic resonance imaging is the modality of choice for neuro-oncologic imaging, neurodegenerative diseases and epilepsy.^2,9^ Multimodal PET/MRI can provide functional information for easier recognition of degeneration patterns specific to Parkinson’s disease, multiple system atrophy, progressive supranuclear palsy and corticobasal degeneration^10^ (Figure 5).

## CONCLUSION

PET/MRI is among the most exciting recent developments in non-invasive hybrid imaging; the combination of anatomical and functional imaging modalities and the use of different tracers to provide comprehensive valuable information. Growing experience with PET/MRI suggests it could become the imaging modality of choice, particularly for assessment of some regions, such as brain, head and neck, liver and pelvis. However, widely accepted clinical indications remain to be determined before conclusive statements regarding the advantages of PET/MRI, over PET/CT can be made.
